# Long-Term Outcome Following Prenatal Diagnosis of Transposition of the Great Arteries

**DOI:** 10.1007/s00246-025-03939-w

**Published:** 2025-07-15

**Authors:** Peter Lillitos, Grace Moriarty, Thomas Witter, Conal Austin, Owen Miller, Gurleen K. Sharland, John M. Simpson, Vita Zidere, Trisha V. Vigneswaran

**Affiliations:** 1 Department of Congenital Heart Disease, Evelina London Children’s Hospital, Guy’s & St Thomas’ NHS Trust, Westminster Bridge Road, London, SE1 7EH UK; 2 School of Biomedical Engineering & Imaging Sciences, King’s College London, London, UK

**Keywords:** Fetus, Transposition of the great arteries, Prenatal diagnosis, Arterial switch operation, Survival, Outcomes, Reintervention, Congenital heart disease

## Abstract

Outcomes following the arterial switch operation (ASO) are well documented. Survival and long-term morbidity for fetuses diagnosed with dextro-transposition of the great arteries (d-TGA) are less reported. We aimed to document survival, reinterventions and morbidity for prenatally diagnosed d-TGA to inform prenatal counseling. Fetuses with d-TGA with or without ventricular septal defect (VSD) diagnosed between 1995 and 2022 at our institution were reviewed. Outcomes were compared to postnatally diagnosed patients undergoing intervention during the same era. Two hundred and seven fetuses were diagnosed resulting in 201 livebirths, 2 intrauterine demise, and 4 pregnancy terminations. There were 137 (68.2%) with isolated d-TGA and 64 (31.8%) with d-TGA-VSD. Median birthweight 3.21 kg (IQR 2.94–3.5 kg), and median birth gestation 38 weeks (IQR 38–39). Preoperative balloon atrial septostomy (BAS) was performed in 126/201(62.7%). No patients died prior to BAS. Three died before ASO following BAS. ASO was performed in 198/201(98.5%) with 45/198(22.7%) having concomitant VSD closure. Thirty-day survival for ASO was 95.5%. Survival over the study period was 186/201(92.5%). During the same period, 91 infants were referred for surgery with postnatal diagnosis, and 90 underwent ASO. There was no significant difference in 30-day or long-term survival following ASO according to timing of diagnosis. Three prenatally diagnosed patients undergoing the ASO were lost to follow up. There was no significant difference in reintervention rates (prenatal: 18/195 (9.2%); postnatal: 12/86 (13.9%) *p* = 0.24). Morbidity in prenatally diagnosed patients included myocardial dysfunction 3/195(1.5%), pulmonary hypertension 1/195(0.5%), supraventricular tachycardia 3/195(1.5%), neurological morbidity 9/195(4.6%), and autism 11/195(5.6%), and none were statistically different to the postnatal group. Survival following prenatal diagnosis of d-TGA is good. In the prenatally diagnosed cohort there were no deaths prior to BAS. Three died after BAS, with one of these deaths attributable to newborn hypoxia between BAS and ASO. Following ASO, most patients survive into third decade following prenatal diagnosis. Reintervention occurred in 9.2%. Neurological and behavioral morbidity affected approximately 1 in 20 patients. Outcomes for those with prenatal diagnosis of d-TGA are comparable with the patients that undergo ASO following postnatal diagnosis, but this excludes patients with a postnatal diangosis who did not survive to cardiac intervention. This data will be helpful for prenatal counseling.

**Supplementary Information:**

The online version contains supplementary material available at 10.1007/s00246-025-03939-w.

## Introduction

Dextro-transposition of the great arteries (d-TGA) is a cyanotic congenital heart lesion with a reported prevalence of 0.2 per 1000 live births [1]. The United Kingdom national cardiac surgical audit data show that for infants with TGA undergoing the arterial switch operation (ASO), prenatal detection has increased from 50% before 2016 [2] to > 80% in the recent years [3]. This owes to improved detection through the National Fetal Anomaly Screening Programme which incorporated the three-vessel and tracheal view in 2015. Prenatal diagnosis of d-TGA has been shown to reduce early postnatal mortality and preoperative morbidity [4–7]. The ASO is now into its fourth decade [8] with excellent 30-day survival outcomes reported [3]. In the current era, most cases are diagnosed prenatally, but there is a paucity of data on long-term outcomes for this cohort. In addition, rates of cardiac reintervention and associated long-term morbidity, including neurodevelopment, have varying reported incidences [9–14]. Therefore, to better inform parental discussion, we sought to analyze our data for outcomes in terms of survival and medium to long-term morbidity for patients prenatally diagnosed with d-TGA.

## Methods

This was a retrospective cohort study of patients prenatally diagnosed with d-TGA and d-TGA-VSD where families were counseled for arterial switch operation, at the Evelina London Children’s Hospital, Guy’s & St Thomas’ NHS Foundation Trust, UK, which is a tertiary pediatric cardiac surgical center. Fetuses diagnosed between 1st January 1995 and 31st December 2022 were identified from the fetal cardiac database (Filemaker Pro, Claris Corp, California) which has been prospectively maintained since 1982. Those with more complex forms of d-TGA were excluded. Prenatal and postnatal records were reviewed. Following prenatal diagnosis of d-TGA or d-TGA-VSD, delivery was planned at our hospital, there was a short period when maternity and pediatric cardiology were on different sites, otherwise there has been geographic co-location of maternity and pediatric cardiology. Onsite delivery was offered to minimize newborn transfers and parent–baby separation.

In addition, all postnatal diagnoses of d-TGA from our congenital heart disease (CHD) network, who had undergone a cardiac intervention (including BAS and ASO) during the same period, were collated from our postnatal database. The primary outcome measure was postnatal survival. Secondary outcomes studied were reinterventions and development of associated morbidity. Patients who emigrated out of the UK were excluded from analyses beyond 30 days and those followed up in another UK center were excluded from long-term morbidity and reintervention analysis as data were unavailable.

The institutional surgical approach to all d-TGA with an antero-posterior arrangement of the great arteries is to switch with Le Compte maneuver. In the less common cases, when there is a true side-by-side arrangement, then Le Compte is not required and the pulmonary bifurcation is moved leftward. The coronary arteries are translocated using a medially based trapdoor incision.

### Statistics

Categorical data were compared using Chi-squared test. Continuous values are summarized as the median (IQR), with comparisons analyzed using the Mann–Whitney *U* test. Kaplan–Meier survival curves demonstrate survival outcomes with the Log-rank test used to compare survival curves where relevant. Statistical significance was defined by a *p* value < 0.05. Statistical analyses were performed using MedCalc® Statistical Software version 22.016 (MedCalc-Software-Ltd, Ostend, Belgium; https://www.medcalc.org; 2023).

### Ethical Approval

This study was classified by our institutional governance department as clinical service evaluation (ref. 15443) using routine collected clinical data and requirement for ethical approval was not required.

## Results

Two hundred and seven fetuses fulfilled the inclusion criteria. Median gestation at diagnosis was 21 weeks (IQR 20–23 weeks). Two fetuses with no other reported comorbidities died in utero: one stillborn near term and one intrauterine demise at 35-week gestation. Four pregnancies (1.9%) were terminated. Of the 201 babies born alive, 137/201 (68.2%) had isolated d-TGA and 64/201 (31.8%) had d-TGA-VSD. No patients with a prenatal diagnosis died prior to BAS.

**Fig. 1:**
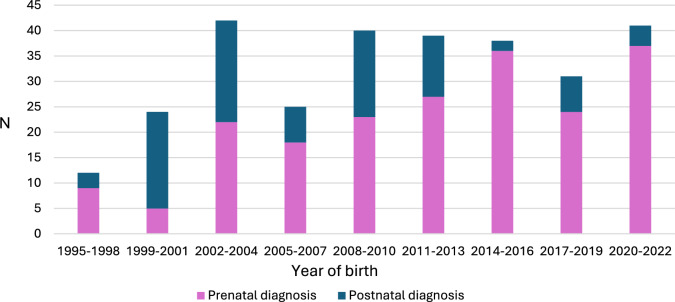
Comparison of patients with prenatally diagnosed d-TGA and those postnatally diagnosed undergoing surgery according to year of birth

**Fig. 2:**
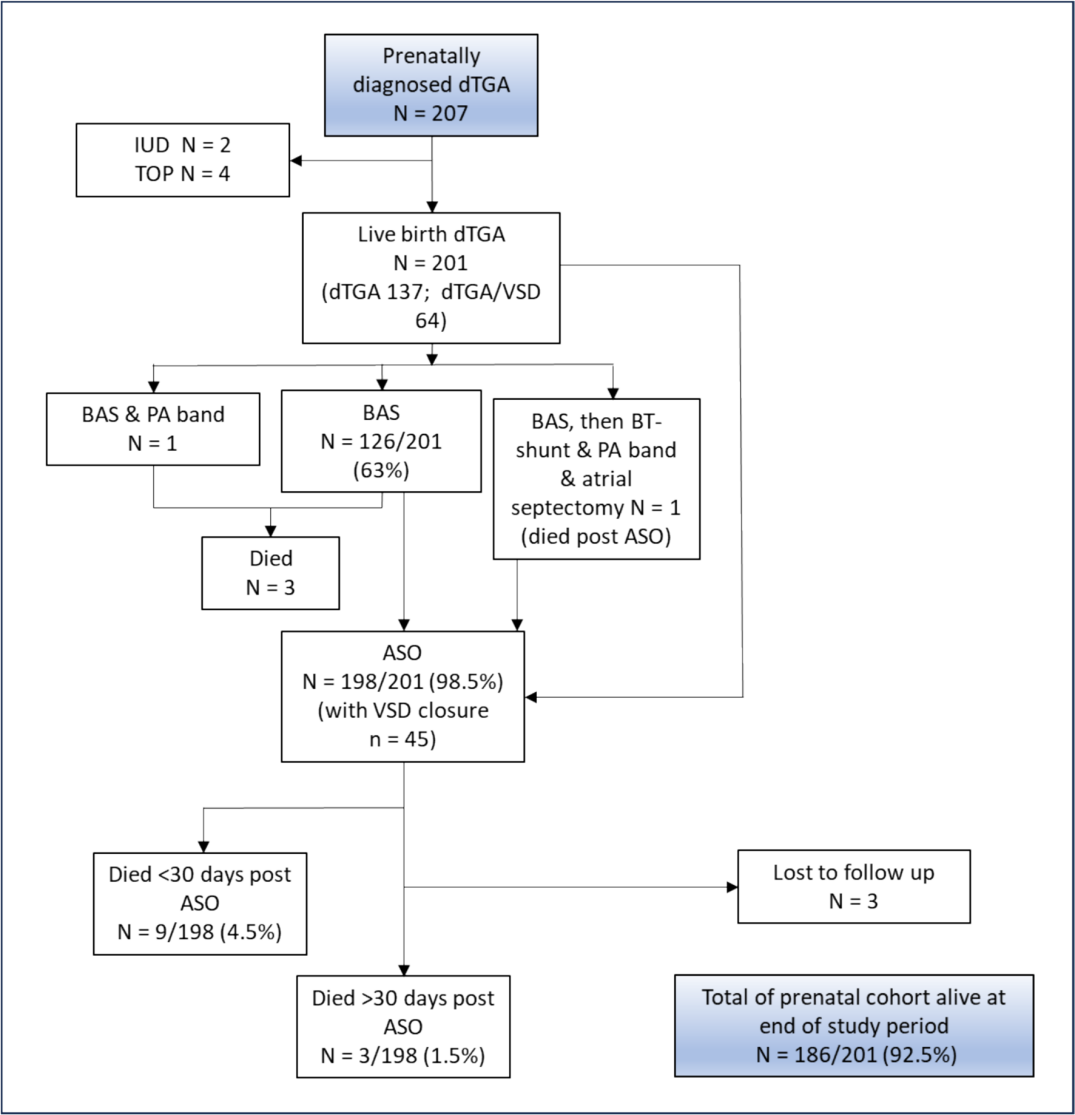
Survival outcomes following fetal diagnosis

During the same era, 109 patients were registered on our cardiac interventions database with a postnatal diagnosis of d-TGA. Eighteen were born outside of our congenital heart network and were excluded, leaving 91 (67 d-TGA, 24 d-TGA-VSD) patients included in analysis. Temporal changes in timing of diagnosis (prenatal vs postnatal) of d-TGA are shown in Fig. 1. The survival outcomes from fetal diagnosis are summarized in Fig. 2. One term patient in the prenatal group died of persistent pulmonary hypertension of the newborn (PPHN) following ASO. This patient was born at 38 weeks weighing over 3 kg and had narcotic abstinence syndrome after birth. In the prenatally diagnosed group, 13 patients had extracardiac anomalies (ECA) and these are summarized in supplementary Table 1. Two patients in the postnatally diagnosed d-TGA group had ECAs: 1 with horseshoe kidney and 1 with trisomy XXY with Goldenhar syndrome. The latter patient died in the early postoperative period following ASO.

### Procedures

The cardiac interventions undertaken in the prenatal and postnatal cohorts are summarized in Table 1. All prenatally diagnosed patients underwent a cardiac procedure. Two-thirds of both groups underwent BAS. In the prenatal group, 65/126 (51.6%) BAS were performed in the first 24 h after birth. In the prenatal group, three patients died between BAS and ASO—one of the complications of prematurity (born 28-week gestation) following an uncomplicated BAS; the second died from sequalae of severe hypoxia secondary to an intact atrial septum following birth. Another patient born prematurely at 31 weeks, weighing < 2 kg and with additional multiple VSDs (and 47XXX karyotype), underwent BAS and PAB but died later from sepsis. In the postnatal group, no patients undergoing BAS died prior to ASO.

**Table 1:** Comparison of characteristics and procedures in the prenatal and postnatal d-TGA cohorts

	Prenatal diagnosis *n* = 201	Postnatal diagnosis *n* = 91	*P* value
Male gender	145 (72.1)	59 (64.8)	NS
Birth weight kg, median (IQR)	3.21 (2.94–3.5)	3.41 (2.96–3.7)	NS
No. of SGA (< 10th centile for GA)	19 (9.5)	13 (14.3)	NS
Median birth gestation, weeks (IQR)	38 (38–39)	40 (40–40)	< 0.0001
Median age (days) at presentation (IQR)		2 (0–16)	
Isolated d-TGA (%)	137 (68.2)	67 (73.6)	NS
d-TGA-VSD (%)	64 (31.8)	24 (26.4)	NS
BAS (%)	126 (62.7)	58 (63.7)	NS
PAB/BTTS (%)	1 (0.5)	3 (3.3)	NS
PAB (%)	1 (0.5)	1 (1.1)	NS
Senning (1st procedure) (%)	0	1 (1.1)	–
ASO (%)	198 (98.5)	90 (98.9)	NS
- with VSD closure	45	21	NS
Median age at BAS, days (IQR)	0 (0–1)	2 (1–16)	< 0.0001
Median age at ASO days (IQR)	8 (6–12)	12 (7–23)	< 0.0001
30-day survival post-ASO surgery	189/198 (95.5%)	86/90 (95.6)	NS
Lost to post-surgical follow-up beyond 30 days	3	4	

*PAB* pulmonary artery band, *BTTS* Blalock–Taussig–Thomas shunt, *SGA* small for gestational age, *ASO *arterial switch operation, *BAS *balloon atrial septostomy

**Fig. 3:**
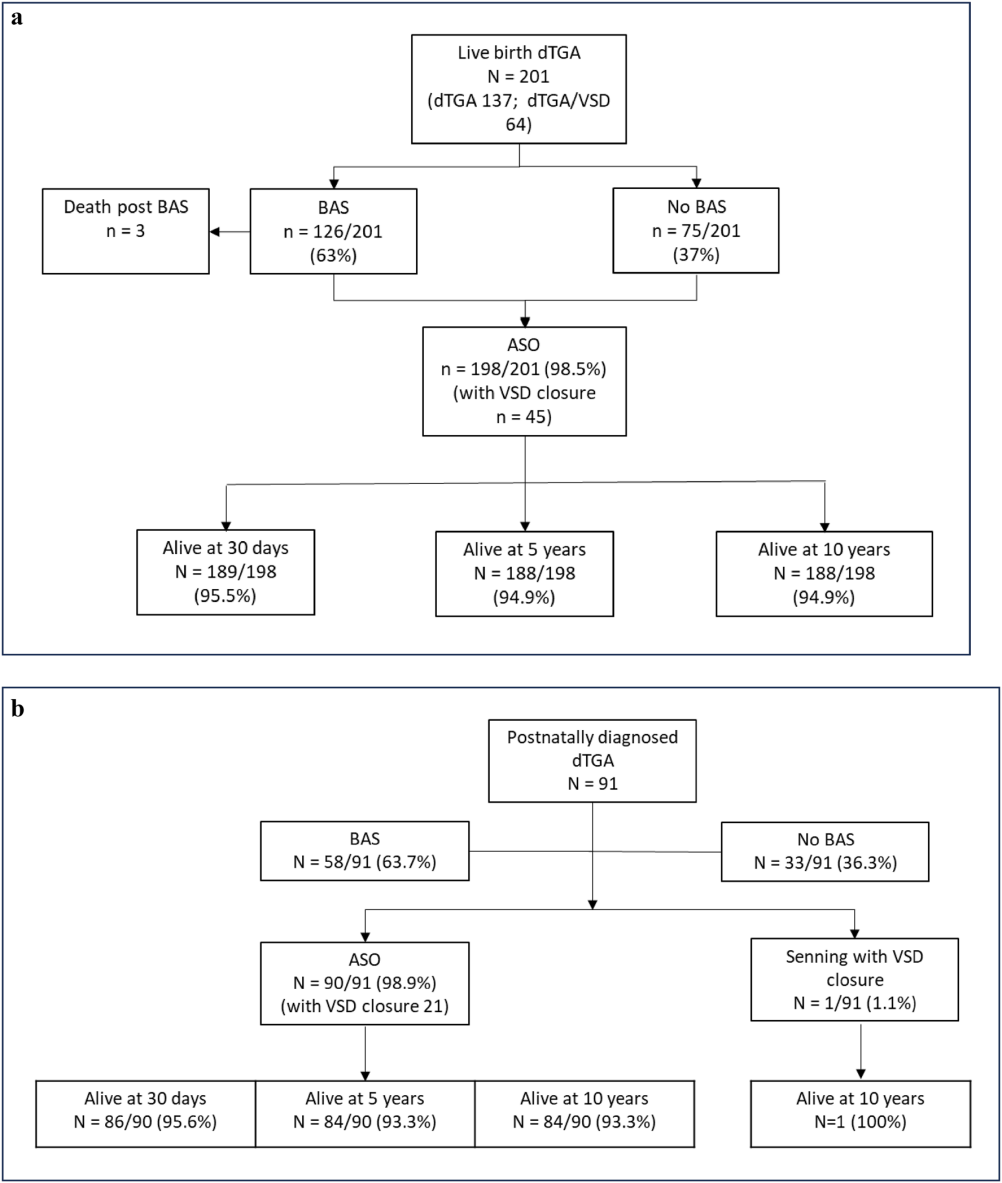
**a** Survival of prenatally diagnosed patients with d-TGA undergoing ASO and survival. **b** Postnatally diagnosed patients undergoing ASO and survival

In the prenatal cohort, ASO was undertaken in 198/201 (98.5%) and of these 45/201 (22.4%) included ASO with VSD closure. In the postnatal group, 90/91 underwent an ASO and one had a Senning operation with VSD closure. In three cases, an initial operation of PAB with Blalock–Taussig–Thomas shunt (BTTS) was undertaken prior to the ASO. Median age at ASO was 8-days (IQR 6–12 days) in the prenatal group and 12 days (IQR 7–23 days) in the postnatal group (*p* < 0.0001). ASO did not take place initially in one prenatally diagnosed infant due to an intraoperative finding of an intramural coronary artery, an atrial septectomy, PAB and BTTS were performed with postoperative extracorporeal membrane oxygenation (ECMO). An ASO was performed at a later time, but death occured secondary to an intracranial hemorrhage. Prenatal and postnatal patients undergoing ASO are shown in Fig. 3a and b, respectively. Of the 13 with ECAs in the prenatal group, 12 underwent ASO and 1 underwent PAB. Of the 2 with ECAs in the postnatal group, all underwent ASO.

### Survival

**Fig. 4:**
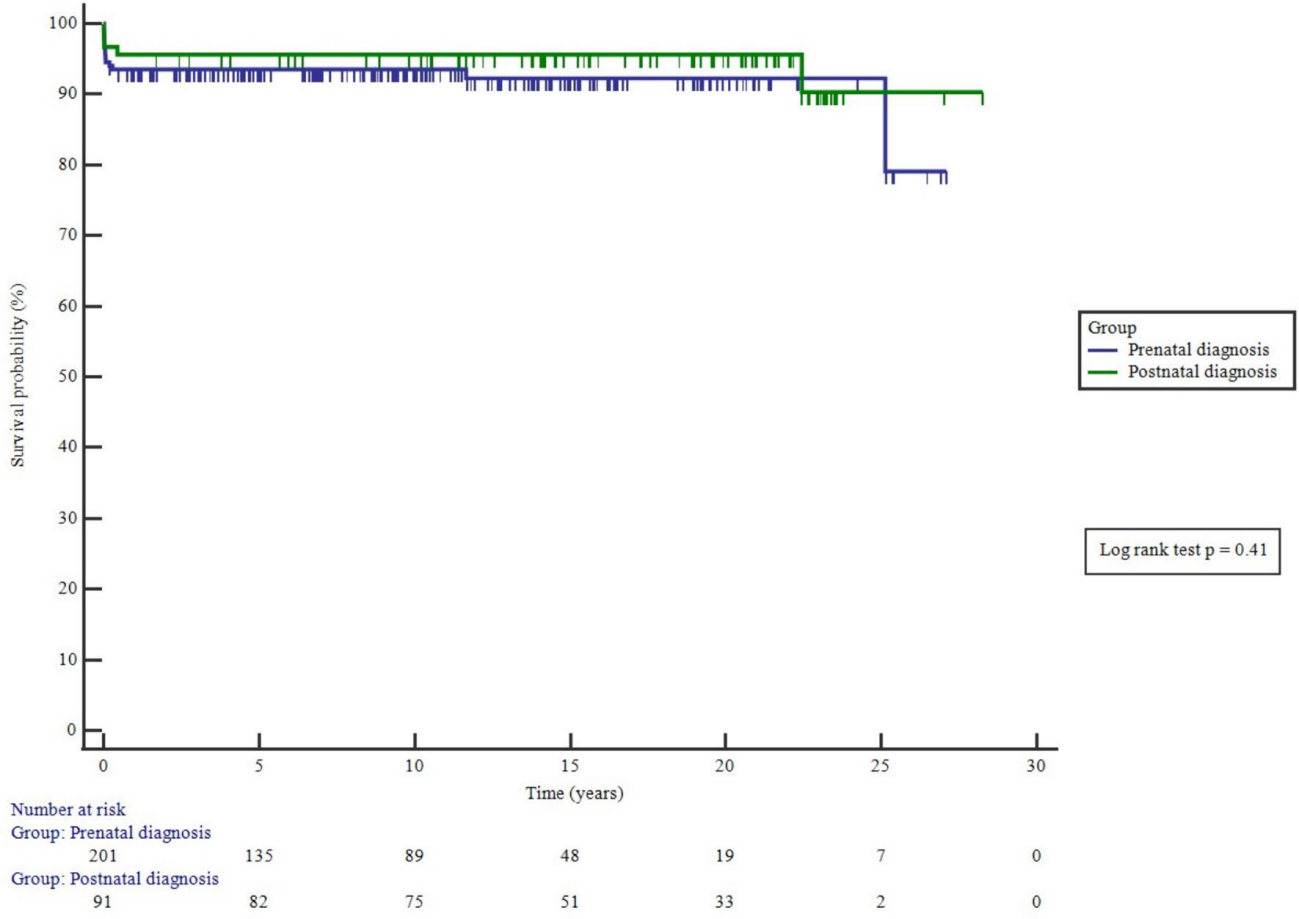
Survival from any first procedure of prenatal and postnatal diagnosed patients with d-TGA

Survival is shown in Table 1. There were no significant differences in survival between male and female patients (*p* = 0.52). There was no significant difference in survival from first procedure (catheter or surgical) in the prenatal and postnatally diagnosed groups (*p* = 0.41), Fig. 4.

The 30-day survival for ASO was the same for both prenatal and postnatal cohorts (95.5%, *p* = 0.97). For the prenatal cohort, 30-day survival after ASO increased by era but did not achieve statistical significance. Survival post-ASO improved after the 1995–2005 era (Table 2). Deaths in those with ECAs were all within 30 days of surgery: 1/13 in the prenatal group and 1/2 in the postnatal group, totaling 2 (13.3%) deaths in all 15 patients with ECAs (prenatal and postnatal groups combined).

**Table 2:** Era survival in the prenatally diagnosed cohort following arterial switch operation

	1995–2005	2006–2015	2016–2022	*P* value
30-day survival	35/39 (89.7%)	85/87 (97.7%)	70/72 (97.2%)	0.09
5-year survival	33/37 (89.2%)	85/87 (97.7%)	69/72 (95.8%)	0.11
10-year survival	33/37 (89.2%)	85/87 (97.7%)	69/72 (95.8%)	0.11

Denominator for each survival period is adjusted for those lost-to-follow-up during study period

### Cardiac Reinterventions

For those that underwent ASO, reinterventions are summarized in Table 3. There were no statistically significant differences in the proportions of patients requiring reintervention between the prenatal and postnatal groups, 18/195 (9.2%) vs 12/87 (13.8%), respectively, *p* = 0.25. Freedom from reintervention for the prenatal group at 1, 5, and 10 to 27 years was 92.7, 92.1, 90.4, and 89.0%, respectively. Those with ECAs in the prenatal group requiring cardiac reintervention was 1/12 (8.3%) vs 17/183 (9.3%) with no ECAs (*p* = 0.91). Postnatally diagnosed d-TGA with ECAs did not undergo reinterventions after their ASO.

**Table 3:** Reintervention post-ASO in those undergoing follow-up

	Prenatal diagnosis *n* = 195	Postnatal diagnosis *n* = 86	*P* value
Median length of follow-up (IQR)	7.6 (6.5–9.4)	14.1 (9.7–18.45)	< 0.0001
Individuals needing reintervention after ASO (%)	18/195 (9.2)	12/86 (13.9)	NS
Type of reintervention			
VSD patch revision	1 (0.5)	1 (1.1)	NS
Wound revision	2 (1.0)	1 (1.1)	NS
Diaphragm plication	1 (0.5)	0	NS
Occlusion of MAPCAs	1 (0.5)	0	NS
Coronary - Revision of coronary reimplantation - Coronary stenosis surgical plasty - Straightening of reimplanted coronary	7 (3.6) 3 3 1	0 – – –	NS
Pulmonary artery interventions (surgical or transcatheter)			NS
Total number of procedures: - Balloon dilatation of MPA - Transannular patch for MPA stenosis - MPA aneurysm revision - Balloon dilatation of branch PS - PA surgical plasty for branch PS - Catheter BPA stent	6 (3.1) 1 1 1 3 3 1	3 (3.5) 1 2	
Aortic valve - Replacement - Repair	2 (1.0) 1 1	3 (3.4) 1 2	NS
Mitral valve cleft closure	0	1 (1.1)	NS
Permanent pacemaker	0	2 (2.3)	0.03
Femoral artery repair (clot occlusion)	0	1 (1.1)	NS

*PAB* pulmonary artery band, *MPA* main pulmonary artery, *PS* pulmonary stenosis, *BPA* branch pulmonary artery, *VSD* ventricular septal defect, *ADHD* attention-deficit hyperactivity disorder, *PPM* permanent pacemaker, *PAB* pulmonary artery band, *BTTS* Blalock–Taussig–Thomas shunt; *MAPCA* major aortopulmonary collaterals, *AVR* aortic valve replacement

There were both early and late coronary artery reinterventions. Two out of the three early coronary interventions occurred in the 1995–2005 era with the remaining one during the 2016-2022 eras. All four patients undergoing late coronary artery reinterventions had abnormal underlying coronary artery anatomy (two with both right and left coronary arteries from sinus 2 and two with retropulmonary circumflex from right coronary artery); all four patients are alive.

### Long-Term Morbidity

Long-term morbidity is summarized in Table 4.

**Table 4:** Morbidities noted following arterial switch operation

	Prenatal diagnosis *n* = 195	Postnatal diagnosis *n* = 86	*P* value
Persistent myocardial dysfunction	4 (2.1)	2 (2.3)	NS
Pulmonary hypertension	1 (0.5)	1 (1.1)	NS
Complete heart block	0	2 (2.3)	0.03
Tachyarrhythmia	3 (1.5)	2 (2.3)	NS
Neurodevelopmental (some patients with > 1 condition):	9 (4.6)	1 (1.2)	NS
- Seizures	6 (3.1)	1 (1.2)	NS
- Learning difficulties or developmental delay	6 (3.1)	0	NS
Autistic spectrum disorder	11 (5.6)	2 (2.3)	NS
Attention-deficit hyperactivity disorder	2 (1.0)	2 (2.3)	NS

There were no significant differences in documented neurological morbidity, autism, or attention-deficit hyperactivity disorder (ADHD) between prenatal and postnatal groups. Patients with an ECA had a significantly higher rate of autism (25% vs 5.4%, *p* = 0.009).

## Discussion

### Main Findings of the Study

Over a 27-year period, there has been an increase in the proportion of prenatally diagnosed patients within our antenatal screening network, with 90.2% of those undergoing a procedure being diagnosed prenatally in the most recent era. This is favorably comparable with the National UK data quoting > 80% prenatal detection of d-TGA [3, 15]. Prenatal diagnosis is associated with low rates of termination of pregnancy (< 2%) and intrauterine demise (< 1%). There were no deaths prior to BAS in the prenatal group. One patient in the prenatal group died of PPHN following ASO. When comparing our prenatally diagnosed cohort to those who underwent ASO following a postnatal diagnosis, the survival following ASO, reintervention rate, and autistic spectrum disorder rate were not different. There were no deaths post-BAS and pre-ASO in the postnatally diagnosed group.

### Prenatal Diagnosis

As with other published studies [11, 16–18], we have also seen an overall increase in the number of patients undergoing ASO operation compared to pre-2000 and possible explanations include increased incidence of d-TGA, transition to ASO as the surgery of choice for patients with d-TGA, and increased early survival of babies born with d-TGA due to improved prenatal and postnatal detection. In the most recent cohort, 90.2% of infants from our CHD network who underwent an ASO had a prenatal diagnosis. As anticipated, this is higher than historic cohorts from other units which have reported 38–50% of ASO with prenatal diagnosis [7, 19–21]. Prenatal diagnosis permits optimisation of delivery planning such that delivery can take place at term if there are no complicating maternal or fetal factors and at the co-located cardiac unit which allows for early access to BAS if required. No patients with a prenatal diagnosis were delivered off-site since 2005. It is well documented that death may occur in newborns with d-TGA due to inadequate mixing of pulmonary and systemic circulations, either due to a restrictive/intact atrial septum, premature closure of the atrial septum [5] or PPHN [22–24]. While approaches to predict which fetuses with d-TGA may have severe hypoxia after birth are used [22, 23, 25–27], these do not identify all patients and delivery at the co-located center is always planned such that emergency access to BAS is available [28]. Our data show that for those fetuses born alive, there were no deaths prior to BAS. There was one death due to sequalae of hypoxia manifesting pre-BAS and two deaths following BAS in premature neonates (28, 31-weeks gestation). No data were available with regards patients who were postnatally diagnosed with d-TGA and whom did not survive to have ASO. Therefore, comparisons of pre-ASO survival between the prenatal and postnatal groups are not possible.

In keeping with other published data [29], there was low prevalence of confirmed underlying genetic abnormalities found on array testing and the occurrence of ECA of 6% is within range observed in other series, between 0.5 and 10% [29, 30]. We found no significant difference in the presence of ECA in the prenatal and postnatal groups. The majority of ECA in our prenatal cohort were diagnosed after birth which highlights the importance of prenatal counseling to inform parents that ECA may only be discovered postnatally.

### Early Survival

We have shown 30-day survival following ASO was over 97% in the modern era and this aligns with nationally observed UK data [3]. Other publications reporting survival using prenatally diagnosed d-TGA cohorts of 72–169 patients show survival to discharge of 97–100% and survival to 1 year of 96–100% [4, 31, 32]. Those reporting outcomes from the point of ASO show survival to discharge 93–99% and to 1 year 92–99% [11, 16, 17, 33]. Consistent with outcomes reported elsewhere [34], most deaths in our cohort occurred early. The trend of improvement in 30-day survival is likely multifactorial: increasing surgical and perioperative intensive care expertise of the ASO, including intraoperative imaging and the availability of ECMO, are likely to have contributed.

Notably, our study only included postnatal diagnoses of d-TGA that underwent an intervention and therefore, this postnatal cohort is a self-selecting group as it does not include those who have not survived to first intervention. This limits a more detailed analysis of the benefits of prenatal diagnosis on survival. A population study by Khoshnood et al. [31] showed no short- to medium-term survival advantage in prenatal compared to postnatally diagnosed d-TGA. Namachivayam et al. recently postulated that poorer postoperative course in those prenatally diagnosed may owe to earlier gestation at birth due to planned births before term for logistical purposes and maternal stress due to knowledge of prenatal diagnosis, which may adversely influence fetal programming [32]. This latter study, however, did not account for maternal factors, fetoplacental complications, site of delivery, and management of relocation across an expansive country to the State providing cardiac surgery or demographic factors which might have contributed to the worse outcome. In our study, we observed that the median gestational age at birth for the prenatal cohort was 38 weeks compared to 40 weeks in the postnatal cohort. There was no difference in the proportion of small for gestational age babies in the prenatal and postnatal cohorts which reflects the shared care approach to pregnancy care within the CHD network and our institutional practice to plan delivery close to 39-week gestation if there are no complicating factors.

### Medium-Long-Term Survival

Over 90% of our cohort have survived into their third decade and this is comparable with other long-term observational studies examining ASO outcomes [16–18]. There was improvement in long-term survival by era which is likely multifactorial, including improved perioperative care and meticulous long-term surveillance for anatomic complications, cardiac function, and signs of coronary artery ischemia. There was no difference in long-term survival between the prenatal cohort and those who presented to our cardiothoracic center and postnatally diagnosed and then underwent BAS or ASO.

### Reintervention

The proportion of patients requiring cardiac reintervention in our prenatal group was 9%, and 5% required reintervention beyond 30 days from ASO. This rate is favorable with recently reported national UK data of 10% at 10 years following ASO [34] and other related studies between 3.5 and 20% [11, 18, 34–36]. Most reintervention procedures were on the right outflow tract and pulmonary branches, consistent with longitudinal data from large ASO follow-up studies [16, 34]. The rate of early coronary complications declined in the recent era. This may owe to the establishment of pre-operative and intraoperative echocardiographic assessment of coronary artery reimplantation. There was a low rate of late coronary reinterventions (1.4% of the whole cohort) which is higher than reported in the United Kingdom [34] and lower than reported in a large Canadian series [37]. All coronary reinterventions were undertaken in those with abnormal coronary arrangements. With more detailed surveillance of the coronary arteries during lifelong follow-up, the identification of coronary pathology is likely to increase over time and hence the rate of later reintervention may rise. Only 1% of prenatal and 3% of postnatal diagnosed d-TGA patients underwent reintervention on their neo-aortic valve or root, and this is consistent with a larger series of d-TGA follow-up data which showed a 96.5% freedom from neo-aortic valve surgery at 20 years after ASO [38]. As in our series, the number of patients which have reached the third decade are few and so longer-term multicentre study is required to assess the reintervention burden.

### Morbidity

Median duration of follow-up was shorter in the prenatal cohort as the rate of prenatal cases was lower than the postnatal cases in the early eras. Reported prevalence of pulmonary hypertension, myocardial dysfunction, and tachyarrhythmias in both prenatal and postnatal groups was low with no significant differences. Though not reaching statistical significance, the prevalence of neurological morbidity was 4 times higher in the prenatal group. The observed proportion in the prenatal group with a diagnosis of autistic spectrum disorder was nearly twice that of the postnatal group and nearly twice the 1–3% reported UK background prevalence [39, 40]. The rate of attention deficit hyperactivity disorder and reported learning difficulties were similar to United Kingdom background population rates [41–43]. The association of d-TGA with neurological morbidity including learning difficulties and neurodevelopmental disorders are well documented with varying reported prevalences [9, 10, 44–47] and etiology is multifactorial [48–50]. While prenatal detection may modify management of those who otherwise may be hypoxic or unstable after delivery and thus more vulnerable to neurological insults, there are other factors to be considered. The higher rates in the prenatal cohort may reflect ascertainment bias, whereby fetal MRI has been undertaken and detected white matter injury and acute ischemic stroke [51]. Furthermore, the MRI appearances of the brain of fetuses with d-TGA resembles premature newborns and may reflect abnormal brain development in utero and together with the earlier birth (38 vs 40 weeks) and slightly earlier timing of the ASO (4 days earlier) might increase later neurodevelopmental morbidity in prenatally diagnosed patients [52, 53]. However, these can be modifiable risk factors with further improvements to obstetric planning. We also found a significantly greater proportion of those with autistic spectrum disorder also had ECA which might relate to an underlying genetic disorder or association of the ECA with abnormal neurodevelopment. The increased prevalence of autistic spectrum disorder in our prenatal group may also relate to parental awareness as it is incorporated into prenatal counseling at the time of diagnosis [54].

## Strengths and Limitations

Our study is a retrospective review of medical records and thus limited by the available data. It was conducted in a center with an electronic fetal cardiology database which was prospectively maintained since its outset in 1982, and pediatric/adult records automatically link to national data which report on deaths. An important limitation is that the postnatally diagnosed cases were identified from our CHD combined surgical and interventional database which only records patients that have undergone a cardiac procedure. This does not include the sickest infants who were admitted to the cardiac unit but did not survive to BAS or ASO or those that died in the community from undiagnosed d-TGA and were only recognized on postmortem. The postnatal cases reported in this study therefore reflect the healthier cohort of postnatally diagnosed cases. With the advent of CHD networks and national datasets in the United Kingdom, there will be the potential to triangulate all neonatal data in future which includes ascertainment of infant deaths in the community from unrecognized CHD which were diagnosed at postmortem. Data regarding coronary artery arrangements were not retrievable. Confirmed neurodevelopmental diagnoses are recorded in our data, but there was no systematic neurodevelopmental and behavioral assessment of patients in our cohort and all diagnoses may not have been recorded.

## Conclusion

Survival following prenatal diagnosis of d-TGA is good. There were no deaths prior to BAS. Three died after BAS, with one of these deaths attributable to newborn hypoxia between BAS and ASO. Following ASO most prenatally diagnosed patients survive into the third decade following prenatal diagnosis. Cardiac reintervention occurred in 9.2%. Neurological and behavioral morbidity affected approximately 1 in 20 patients. Outcomes for those with prenatal diagnosis of d-TGA are comparable with the patients that undergo ASO following postnatal diagnosis, but this excludes patients with a postnatal diangosis who did not survive to cardiac intervention. This data will be helpful for prenatal counseling.

## Supplementary Information

Below is the link to the electronic supplementary material.

Supplementary file1 (DOCX 23 KB)

## Data Availability

Data is provided within the manuscript or supplementary information files. The data that support the findings of this study are available from the corresponding author upon reasonable request.

## Abbreviations

ASO: Arterial switch operation
d-TGA: Dextro-transposition of the great arteries
VSD: Ventricular septal defect
BAS: Balloon atrial septostomy
PAB: Pulmonary artery band
BTTS: Blalock–Taussig–Thomas shunt
ECMO: Extracorporeal membrane oxygenation
PPHN: Persistent pulmonary hypertension of the newborn
CHD: Congenital heart disease
