# Supplementary material for: Long-Term Outcome Following Prenatal Diagnosis of Transposition of the Great Arteries
Source: Pediatr Cardiol. 2025 Jul 15;47(4):1591–601. doi: 10.1007/s00246-025-03939-w (PMC12946309; doi:10.1007/s00246-025-03939-w)
Supplement: Supplementary file 1 — Supplementary file1 (DOCX 23 KB) [file 246_2025_3939_MOESM1_ESM.docx]

Supplementary table. 1. Summary of extracardiac anomalies in 13 prenatally diagnosed patients

| Extracardiac anomalies (ECA) in the prenatally diagnosed d-TGA patients | Timing of ECA diagnosis |
| --- | --- |
| Spinal dysraphism, leg hemi-atrophy, bowel/bladder anomalies | After birth |
| Hemivertebrae | After birth |
| Renal pelvic dilatation | After birth |
| Duodenal atresia  Dysplastic ear/hemivertebrae/VACTERL | Before birth  After birth |
| Hypospadias | After birth |
| Congenital cervical spine fusion | After birth |
| Pre-auricular tags and hearing loss | After birth |
| Hydrocephalus, Chiari 1 malformation, syringomyelia, VACTERL association (absent left thumb, abnormal vertebrate), left multicystic dysplastic kidney. Normal chromosomal analysis and array. | After birth |
| Multicystic dysplastic kidney | After birth |
| Choanal atresia, hemivertebra | After birth |
| Coloboma, pelvic kidney, no genetic cause found | After birth |
| Cleft lip, CHARGE syndrome | After birth |
| 47 XXX and Goldenhar syndrome | Before birth |
